# Searching and Mining Trillions of Time Series Subsequences under Dynamic Time Warping

**DOI:** 10.1145/2339530.2339576

**Published:** 2012-08

**Authors:** Thanawin Rakthanmanon, Bilson Campana, Abdullah Mueen, Gustavo Batista, Brandon Westover, Qiang Zhu, Jesin Zakaria, Eamonn Keogh

**Affiliations:** UC Riverside; UC Riverside; UC Riverside; University of São Paulo; Brigham and Women’s Hospital; UC Riverside; UC Riverside; UC Riverside

**Keywords:** Algorithm, Experimentation, Time series, Similarity Search, Lower Bounds

## Abstract

Most time series data mining algorithms use similarity search as a core subroutine, and thus the time taken for similarity search is *the* bottleneck for virtually all time series data mining algorithms. The difficulty of scaling search to large datasets largely explains why most academic work on time series data mining has plateaued at considering a few millions of time series objects, while much of industry and science sits on billions of time series objects waiting to be explored. In this work we show that by using a combination of four novel ideas we can search and mine truly massive time series for the first time. We demonstrate the following extremely unintuitive fact; in large datasets we can exactly search under DTW much more quickly than the current state-of-the-art *Euclidean distance* search algorithms. We demonstrate our work on the largest set of time series experiments ever attempted. In particular, the largest dataset we consider is larger than the combined size of all of the time series datasets considered in all data mining papers ever published. We show that our ideas allow us to solve higher-level time series data mining problem such as motif discovery and clustering at scales that would otherwise be untenable. In addition to mining massive datasets, we will show that our ideas also have implications for real-time monitoring of data streams, allowing us to handle much faster arrival rates and/or use cheaper and lower powered devices than are currently possible.

## 1. INTRODUCTION

Time series data is pervasive across almost all human endeavors, including medicine, finance, science and entertainment. As such, it is hardly surprising that time series data mining has attracted significant attention and research effort. Most time series data mining algorithms require similarity comparisons as a subroutine, and in spite of the consideration of dozens of alternatives, there is increasing evidence that the classic Dynamic Time Warping (DTW) measure is the best measure in most domains [6].

It is difficult to overstate the ubiquity of DTW. It has been used in robotics, medicine [5], biometrics, music/speech processing [1][27][41], climatology, aviation, gesture recognition [3][38], user interfaces [16][22][29][38], industrial processing, cryptanalysis [7], mining of historical manuscripts [15], geology, astronomy [20][31], space exploration, wildlife monitoring, etc.

As ubiquitous as DTW is, we believe that there are thousands of research efforts that would like to use DTW, but find it too computationally expensive. For example, consider the following: “*Ideally, dynamic time warping would be used to achieve this, but due to time constraints…*” [5]. Likewise, [3] bemoans DTW is “*still too slow for gesture recognition systems*”, and [1] notes, even “*a 30 fold speed increase may not be sufficient for scaling DTW methods to truly massive databases*.” As we shall show, our subsequence search suite of four novel ideas (called the *UCR suite*) removes all of these objections. We can reproduce all the experiments in all these papers in well under a second.

We make an additional claim for our UCR suite which is almost certainly true, but hard to prove, given the variability in how search results are presented in the literature. We believe our exact DTW sequential search is much faster than any current *approximate* search or exact *indexed* search. In a handful of papers the authors *are* explicit enough with their experiments to see this is true. Consider [28], which says it can answer queries of length 1,000 under DTW with 95% accuracy, in a random walk dataset of one million objects in 5.65 seconds. We can *exactly* search this dataset in 3.8 seconds (on a very similar machine). Likewise, a recent paper that introduced a novel inner product based DTW lower bound greatly speeds up exact subsequence search for a wordspotting task in speech. The authors state: “*the new DTW-KNN method takes approximately 2 minutes*” [41]; however, we can reproduce their results in less than a second. An influential paper on gesture recognition on multi-touch screens laments that “*DTW took 128.26 minutes to run the 14,400 tests for a given subject’s 160 gestures*” [38]. However, we can reproduce these results in under 3 seconds.

### 1.1 A Brief Discussion of a Trillion

Since we use the word “trillion” in this work and to our knowledge, it has never appeared in a data mining/database paper, we will take the time to briefly discuss this number. By a trillion, we mean the short scale version of the word [14], one million million, or 10^12^, or 1,000,000,000,000.

If we have a single time series *T* of length one trillion, and we assume it takes eight bytes to store each value, it will require 7.2 terabytes to store. If we sample a electrocardiogram at 256Hz, a trillion data points would allow us to record 123 years of data, every single heartbeat of the longest lived human [37].

A time series of length one trillion is a very large data object. In fact, it is more than all of the time series data considered in all papers ever published in all data mining conferences *combined*. This is easy to see with a quick back-of-the-envelope calculation. Up to 2011 there have been 1,709 KDD/SIGKDD papers (including industrial papers, posters, tutorial/keynote abstracts, etc. [9]). If *every* such paper was on time series, and *each* had looked at five hundred million objects, this would still not add up to the size of the data we consider here). However, the largest time series data considered in a SIGKDD paper was a “mere” one hundred million objects [35].

As large as a trillion is, there are thousands of research labs and commercial enterprises that have this much data. For example, many research hospitals have trillions of data points of EEG data, NASA Ames has tens of trillions of datapoints of telemetry of domestic flights, the Tennessee Valley Authority (a power company) records a trillion data points every four months, etc.

### 1.2 Explicit Statement of our Assumptions

Our work is predicated on several assumptions that we will now enumerate and justify.

#### 1.2.1 Time Series Subsequences must be Normalized

In order to make meaningful comparisons between two time series, both must be normalized. While this may seem intuitive, and was explicitly empirically demonstrated a decade ago in a widely cited paper [19], many research efforts do not seem to realize this. This is critical because some speedup techniques *only* work on the un-normalized data; thus, the contributions of these research efforts may be largely nullified [8][28].

**Figure 1:**
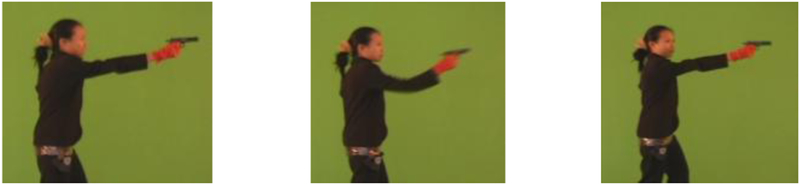
Screen captures from the original video from which the Gun/NoGun data was culled. The center frame is the original size; the left and right frames have been scaled by 110% and 90% respectively. While these changes are barely perceptible, they double the error rate if normalization is not used. (Video courtesy of Dr. Ratanamahatana)

To make this clearer, let us consider the classic Gun/NoGun classification problem which has been in the public domain for nearly a decade. The data, which as shown in Figure 1.*center* is extracted from a video sequence, *was* Z-normalized. The problem has a 50/150 train/test split and a DTW one-nearest-neighbor classifier achieves an error rate of 0.087.

Suppose the data had *not* been normalized. As shown in Figure 1.*left* and Figure 1.*right*, we can simulate this by adding a tiny amount of scaling/offset to the original video. In the first case we randomly change the *offset* of each time series by ± 10%, and in the second case we randomly change the *scale* (amplitude) by ± 10%. The new one-nearest-neighbor classifier error rates, averaged over 1,000 runs, are 0.326 and 0.193, respectively, significantly worse than the normalized case.

It is important to recognize that these tiny changes we made are completely dwarfed by changes we might expect to see in a real world deployment. The apparent *scale* can be changed by the camera zooming, by the actor standing a little closer to the camera, or by an actor of a different height. The apparent *offset* can be changed by this much by the camera tilt angle, or even by the actor wearing different shoes.

While we did this experiment on a visually intuitive example, all forty-five datasets in the UCR archive increase their error rate by at least 50% if we vary the offset and scale by just ± 5%.

It is critical to avoid a common misunderstanding. We must normalize *each* subsequence before making a comparison, it is not sufficient to normalize the entire dataset.

#### 1.2.2 Dynamic Time Warping is the Best Measure

It has been suggested many times in the literature that the problem of time series data mining scalability is only due to DTW’s oft-touted lethargy, and that we could solve this problem by using some other distance measure. As we shall later show, this is not the case. In fact, as we shall demonstrate, our optimized DTW search is much faster than all current *Euclidean* distance searches. Nevertheless, the question remains, is DTW the right measure to speed up? Dozens of alternative measures have been suggested. However, recent empirical evidence strongly suggests that none of these alternatives routinely beats DTW. When put to the test on a collection of forty datasets, the very *best* of these measures are sometimes a little better than DTW and sometimes a little worse [6]. In general, the results are consistent with these measures being minor variants or “flavors” of DTW (although they are not typically presented this way). In summary, after an exhaustive literature search of more than 800 papers [6], we are not aware of any distance measure that has been shown to outperform DTW by a statistically significant amount on reproducible experiments [6][19]. Thus, DTW is *the* measure to optimize (recall that DTW subsumes Euclidean distance as a special case).

#### 1.2.3 Arbitrary Query Lengths cannot be Indexed

If we know the length of queries ahead of time we can mitigate at least some of the intractability of search by indexing the data [2][11][35]. Although to our knowledge no one has built an index for a trillion real-valued objects (Google only indexed a trillion webpages as recently as 2008), perhaps this could be done.

However, what if we do not know the length of the queries in advance? At least two groups have suggested techniques to index arbitrary length queries [18][23]. Both methods essentially build multiple indexes of various lengths, and at query time search the shorter and longer indexes, “interpolating” the results to produce the nearest neighbor produced by a virtual index of the correct length. This is an interesting idea, but it is hard to imagine it is the answer to our problem. Suppose we want to support queries in the range of, say, 16 to 4096. We must build indexes that are not too different in size, say Multindex-lengths = {16, 32, 64, .., 1024, 2048, 4096}^1^. However, for time series data the index is typically about one-tenth the size of the data [6][18]. Thus, we have doubled the amount of disk space we need. Moreover, if we are interested in tackling a trillion data objects we clearly cannot fit *any* index in the main memory, much less all of them, or any two of them.

There is an underappreciated reason why this problem is so hard; it is an implication of the need for normalization discussed above. Suppose we have a query *Q* of length 65, and an index that supports queries of length 64. We search the index for *Q*_[1:64]_ and find that the best match for it has a distance of, say, 5.17. What can we say about the best match for the full *Q*? The answer is surprisingly little: 5.17 is neither an upper bound nor a lower bound to the best match for *Q*. This is because we must renormalize the subsequence when moving from *Q*_[1:64]_ to the full *Q*. If we do not normalize any data, the results are meaningless (cf. Section 1.2.1), and the idea *might* be faster than sequential search. However, if we normalize the data we get so little information from indexes of the wrong length that we are no better off than sequential search.

In summary, there are no known techniques to support similarity search of arbitrary lengths once we have datasets in the billions.

#### 1.2.4 There Exists Data Mining Problems that we are Willing to Wait Some Hours to Answer

This point is almost self-evident. If a team of entomologists has spent three years gathering 0.2 trillion datapoints [35], or astronomers have spent billions dollars to launch a satellite to collect one trillion datapoints of star-light curve data per day [20], or a hospital charges $34,000 for a daylong EEG session to collect 0.3 trillion datapoints (c.f. Section 5.2) [26], then it is not unreasonable to expect that these groups would be willing to spend hours of CPU time to glean knowledge from their data.

## 2. RELATED WORK

Our review of related work on time series indexing is necessarily superficial, given the vast amount of work on the topic and page limits. Instead, we refer the interested reader to two recent papers [6][28], which have comprehensive reviews of existing work. It has now become common (although not yet *routine*) to see papers indexing/mining datasets with millions of objects. For example, Jegou et al. have demonstrated very fast approximate main memory search of 10 million images [17]. However, this work and much of the current work that addresses multi-million object datasets focus on *approximate* search, whereas we are only considering *exact* search here. Moreover, we are interested in datasets which are five to six orders of magnitude larger than anything else considered in the literature [6]. Thus, comparisons to related work are very difficult to do meaningfully.

## 3. BACKGROUND AND NOTATIONS

### 3.1 Definitions and Notations

We begin by defining the data type of interest, time series:

**Definition 1:** A *Time Series T* is an ordered list: *T*=*t*_1_,*t*_2_,…,*t*_*m*_. While the source data is one long time series, we ultimately wish to compare it to shorter regions called *subsequences*:

**Definition 2:** A *subsequence T*_*i*,*k*_ of a time series *T* is a shorter time series of length *k* which starts from position *i*. Formally, *T*_*i*,*k*_ = *t*_*i*_*,t*_*i+1*_,..*,t*_*i*+k−1_, 1≤ *i* ≤ *m*−*k*+1. Where there is no ambiguity, we may refer to subsequence *T*_*i*,*k*_ as *C*, as in a *C*andidate match to a query *Q*. We denote |*Q*| as *n*.

**Definition 3:** The Euclidean distance (ED) between *Q* and *C*, where |*Q*| =|*C*|, is defined as:

$$ED\left(Q,C\right)=\sqrt{\sum_{i=1}^{n}{\left(q_{i}-c_{i}\right)}^{2}}$$

**Figure 2:**
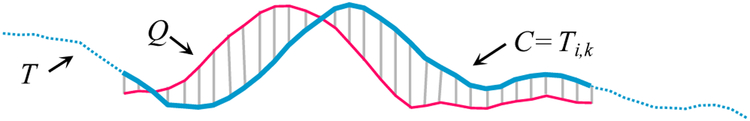
A long time series *T* can have a subsequence *T*_*i,k*_ extracted and compared to a query *Q* under the Euclidean distance, which is simply the square root of the sum of the squared hatch line lengths

We illustrate these definitions in Figure 2.

**Figure 3:**
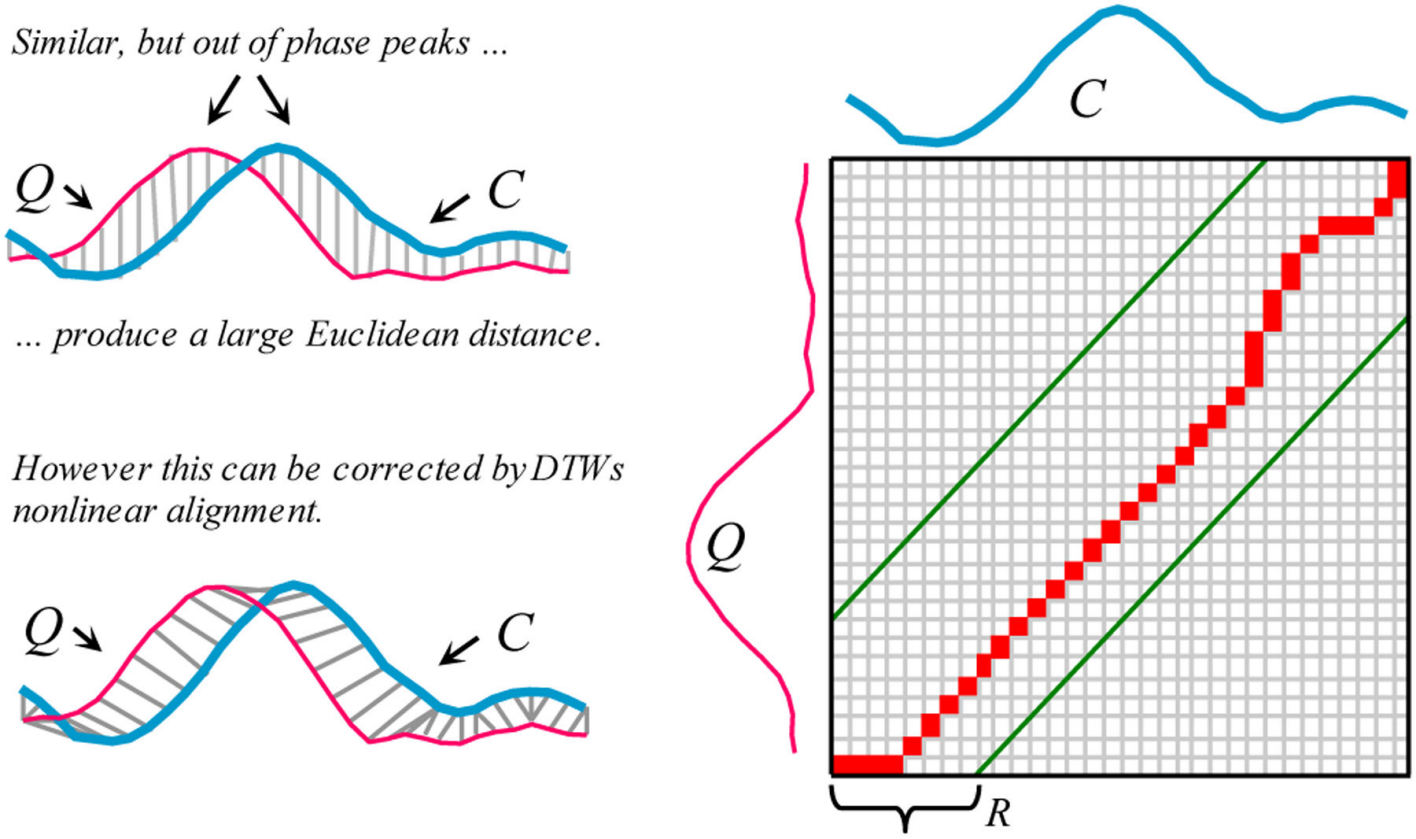
*left*) Two time series which are similar but out of phase. *right*) To align the sequences we construct a warping matrix, and search for the optimal warping path (red/solid squares). Note that Sakoe-Chiba Band with width *R* is used to constrain the warping path

The Euclidean distance, which is a one-to-one mapping of the two sequences, can be seen as a special case of DTW, which allows a one-to-many alignment, as illustrated in Figure 3.

To align two sequences using DTW, an *n*-by-*n* matrix is constructed, with the (*i*^th^, *j*^th^) element of the matrix being the Euclidean distance *d*(*q*_i_, *c*_j_) between the points *q*_i_ and *c*_j_.

A warping path *P* is a contiguous set of matrix elements that defines a mapping between *Q* and *C*. The *t*^th^ element of *P* is defined as *p*_t_ = (*i*, *j*)_t_ so we have:

$$P=p_{1},p_{2},\;\ldots ,p_{\text{t}},\;\ldots ,p_{\text{T}}\;n\leq \text{T}\leq 2n-1$$

The warping path that defines the alignment between the two time series is subject to several constraints. For example, the warping path must start and finish in diagonally opposite corner cells of the matrix, the steps in the warping path are restricted to adjacent cells, and the points in the warping path must be monotonically spaced in time. In addition, virtually all practitioners using DTW also constrain the warping path in a global sense by limiting how far it may stray from the diagonal [6][28]. A typical constraint is the Sakoe-Chiba Band which states that the warping path cannot deviate more than *R* cells from the diagonal [6][28][32].

## 4. ALGORITHMS

### 4.1 Known Optimizations

We begin by discussing previously known optimizations of sequential search under ED and/or DTW.

#### 4.1.1 Using the Squared Distance

Both DTW and ED have a square root calculation. However, if we omit this step, it does not change the relative rankings of nearest neighbors, since both functions are monotonic and concave. Moreover, the absence of the square root function will make later optimizations possible and easier to explain. Note that this is only an internal change in the code; the user can still issue range queries with the original units, as the code simply internally squares the desired value, does the search, and after finding the qualifying objects, takes the square root of the distances for the qualifying objects and presents the answers to the user.

Where there is no ambiguity below, we will still use ‘DTW’ and ‘ED’; however, the reader may assume we mean the squared versions of them.

#### 4.1.2 Lower Bounding

**Figure 4:**
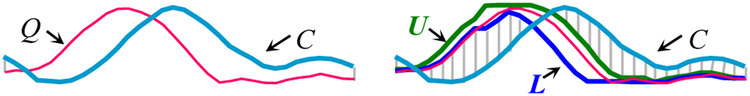
*left*) The *LB_*_*Kim*_*FL* lower bound is *O*(1) and uses the distances between the First (Last) pair of points from *C* and *Q* as a lower bound. It is a simplification of the original *LB_*_*Kim*_ [21]. *right*) The *LB_*_*Keogh*_ lower bound is *O*(*n*) and uses the Euclidean distance between the candidate sequence *C* and the closer of {*U*,*L*} as a lower bound

A classic trick to speed up sequential search with an expensive distance measure such as DTW is to use a cheap-to-compute lower bound to prune off unpromising candidates [6][20]. Figure 4 shows two such lower bounds, one of which we have modified.

The original definition of *LB_*_*Kim*_ also uses the distances between the maximum values from both time series and the minimum values between both time series in the lower bound, making it *O*(*n*). However, for *normalized* time series these two extra values tend to be tiny and it does not pay to compute them, and ignoring them allows the bound to be *O*(1), a fact we will exploit below. The *LB_*_*Keogh*_ bound is well-documented elsewhere, for brevity we ask the unfamiliar reader to refer to [11][20][6] for a review.

#### 4.1.3 Early Abandoning of ED and LB__Keogh_

**Figure 5:**
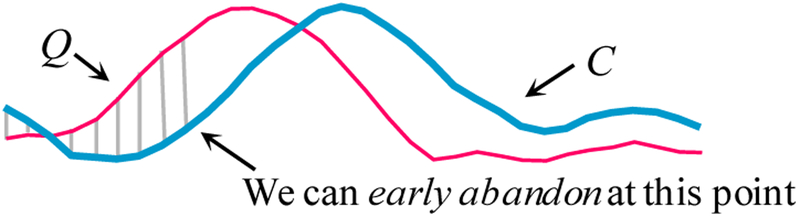
An illustration of ED *early abandoning*. We have a *best-so-far* value of *b*. After incrementally summing the first nine (of thirty-two) individual contributions to the ED we have exceeded *b*, thus it is pointless to continue the calculation [20]

During the computation of the Euclidean distance or the *LB_*_*Keogh*_ lower bound, if we note that the current sum of the squared differences between each pair of corresponding datapoints exceeds the *best-so-far*, then we can stop the calculation, secure in the knowledge that the exact distance or lower bound, had we calculated it, would have exceeded the *best-so-far*, as in Figure 5.

#### 4.1.4 Early Abandoning of DTW

**Figure 6:**
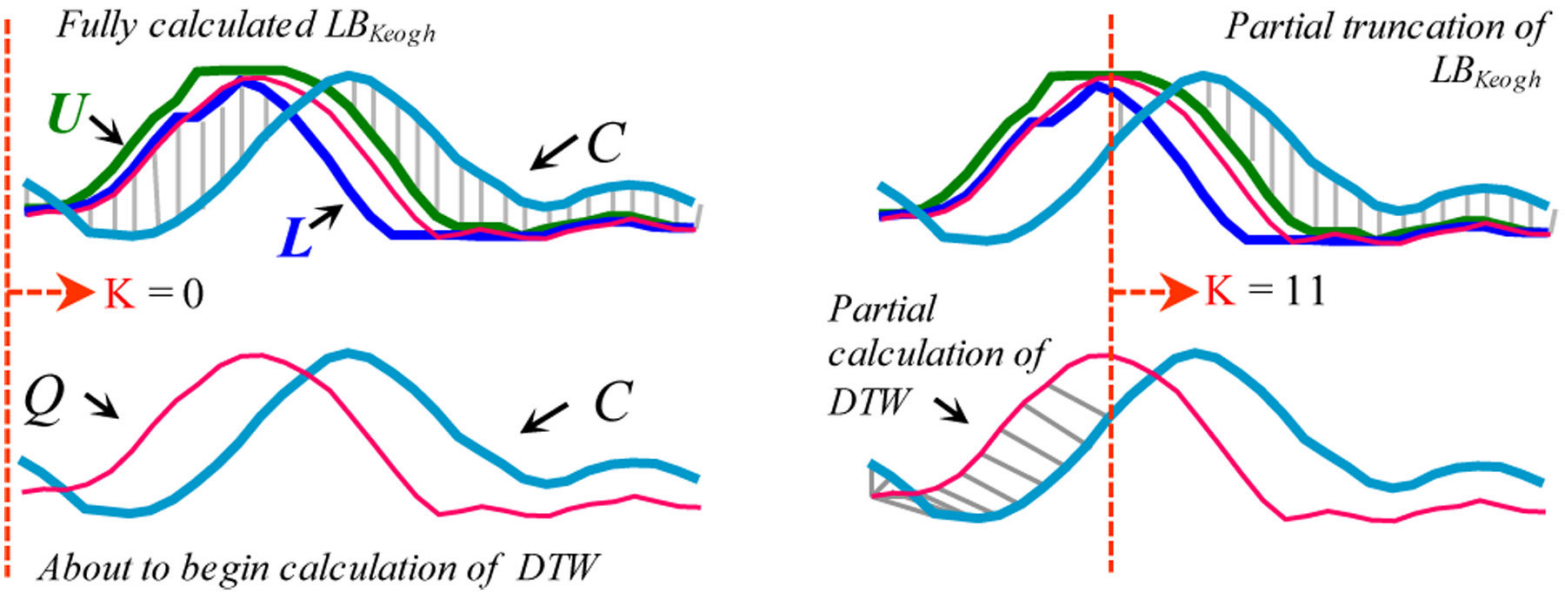
*left*) At the top we see a completed *LB_*_*Keogh*_ calculation, and below it we are about to begin a full DTW calculation. *right*) We can imagine the orange/dashed line moving from left to right. If we sum the *LB_*_*Keogh*_ contribution from the right of dashed line (*top*) and the partial (incrementally calculated) DTW contribution from the left side of the dashed line (*bottom*), this is will be a lower bound to DTW(*Q*,*C*)

If we have computed a full *LB_*_*Keogh*_ lower bound, but we find that we must calculate the full DTW, there is still one trick left up our sleeves. We can incrementally compute the DTW from left to right, and as we incrementally calculate from 1 to K, we can sum the *partial* DTW accumulation with the *LB_*_*Keogh*_ contribution from K+1 to *n*. Figure 6 illustrates this idea.

This sum of DTW(Q_1:K_,C_1:K_) + *LB_*_*Keogh*_(Q_K+1:*n*_,C_K+1:*n*_) is a lower bound to the true DTW distance (i.e., DTW(Q_1:*n*_,C_1:*n*_)). Moreover, with careful implementation the overhead costs are negligible. If at any time this lower bound exceeds the *best-so-far* distance we can admissibly stop the calculation and prune this *C*.

#### 4.1.5 Exploiting Multicores

It is important to note that while we can get essentially linear speedup using multicores, the *software* improvements we will present in the next section completely dwarf the improvements gained by multicores. As a concrete example, a recent paper shows that a search of a time series of length 421,322 under DTW takes “*3 hours and 2 minutes on a single core. The* (8-core version) *was able to complete the computation in 23 minutes*” [34]. However, using our ideas, we can search a dataset of this size in just under one second on a single core. Nevertheless, as it is simple to port to the now ubiquitous multicores, we consider them below.

### 4.2 Novel Optimizations: The UCR Suite

We are finally in a position to introduce our four original optimizations of search under ED and/or DTW.

#### 4.2.1 Early Abandoning Z-Normalization

To the best of our knowledge, no one has ever considered optimizing the *normalization* step. This is surprising, since it takes slightly longer than computing the Euclidean distance itself.

Our insight here is that we can interleave the early abandoning calculations of Euclidean distance (or *LB_*_*Keogh*_) with the online Z-normalization. In other words, as we are incrementally computing the Z-normalization, we can also incrementally compute the Euclidean distance (or *LB_*_*Keogh*_) of the same datapoint. Thus, if we can early abandon, we are pruning not just distance calculation steps as in Section 4.1.3, but also *normalization* steps.

Recall that the mean and standard deviation of a sample can be computed from the sums of the values and their squares. Therefore, it takes only one scan through the sample to compute the mean and standard deviation, using the equations below.

$$\mu =\frac{1}{m}\sum x_{i}\;\sigma^{2}=\frac{1}{m}\sum x_{i}^{2}-\mu^{2}$$

In similarity search, every subsequence needs to be normalized before it is compared to the query (c.f. Section 1.2.1). The mean of the subsequence can be obtained by keeping two running sums of the long time series which have a lag of exactly *m* values. The sum of squares of the subsequence can be similarly computed. The formulas are given below for clarity.

$$\mu =\frac{1}{m}\left(\sum_{i=1}^{k}x_{i}-\sum_{i=1}^{k-m}x_{i}\right)\;\sigma^{2}=\frac{1}{m}\left(\sum_{i=1}^{k}x_{i}^{2}-\sum_{i=1}^{k-m}x_{i}^{2}\right)-\mu^{2}$$

The high-level outline of the algorithm is presented in Table 1.

Note the online normalization in line 11 of the algorithm, which allows the early abandoning of the distance computation in addition to the normalization.

In the above algorithm, we use a circular buffer (X) to store the current subsequence being compared with the query *Q*.

One potential problem of this method of maintaining the statistics is the accumulation of the floating-point error [13]. The effect of such error accumulation is more profound if all of the numbers are positive, as in our case with sum of squares. With the “mere” millions of datapoints the rest of the community has dealt with this effect is negligible, however when dealing with billions of datapoints it *will* affect the answer. Our simple solution is that once every one million subsequences, we force a complete Z-normalization to “flush out” any accumulated error.

#### 4.2.2 Reordering Early Abandoning

In the previous section, we saw that the idea of early abandoning discussed in Section 4.1.3 can be generalized to the Z-normalization step. In both cases, we assumed that we incrementally compute the distance/normalization from left to right. Is there a better ordering?

**Figure 7:**
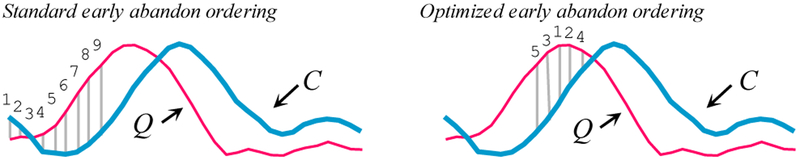
*left*) ED *early abandoning*. We have a *best-so-far* value of *b*. After incrementally summing the first *nine* individual contributions to the ED, we have exceeded *b*; thus, we abandon the calculation. *right*) A different ordering allows us to abandon after just *five* calculations

Consider Figure 7.*left*, which shows the normal left-to-right ordering in which the early abandoning calculation proceeds. In this case *nine* of the thirty-two calculations were performed before the accumulated distance exceeded *b* and we could abandon. In contrast, Figure 7.*right* uses a different ordering and was able to abandon earlier, with just *five* of the thirty-two calculations.

This example shows what is obvious: on a query-by-query basis, different orderings produce different speedups. However, we want to know if there is a *universal* optimal ordering that we can compute *in advance*. This seems like a difficult question because there are *n*! possible orderings to consider.

We conjecture that the universal optimal ordering is to sort the indices based on the absolute values of the Z-normalized *Q*. The intuition behind this idea is that the value at *Q*_i_ will be compared to many *C*_i_’s during a search. However, for subsequence search, with Z-normalized candidates, the distribution of many *C*_i_’s will be Gaussian, with a mean of zero. Thus, the sections of the query that are farthest from the mean, zero, will *on average* have the largest contributions to the distance measure.

To see if our conjecture is true we took the heartbeat discussed in Section 5.4 and computed its full Euclidean distance to a million other randomly chosen ECG sequences. With the conceit of hindsight we computed what the best ordering *would* have been. For this we simply take each *C*_i_ and sort them, largest first, by their sum of their contributions to the Euclidean distance. We compared this *empirically* optimal ordering with our predicted ordering (sorting the indices on the absolute values of *Q*) and found the rank correlation is 0.999. Note that we can use this trick for both ED and *LB_*_*Keogh*_, and we can use it in conjunction with the early abandoning Z-normalization technique (Section 4.2.1).

#### 4.2.3 Reversing the Query/Data Role in LB__Keogh_

**Figure 8:**
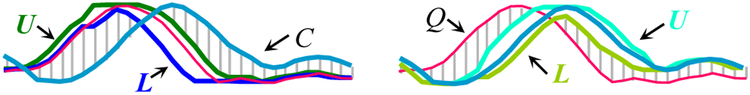
*left*) Normally the *LB_*_*Keogh*_ envelope is built around the query (see also Figure 4.*right*), and the distance between *C* and the closer of {*U*,*L*} acts as a lower bound. *right*) However, we can reverse the roles such that the envelope is built around *C* and the distance between *Q* and the closer of {*U*,*L*} is the lower bound

Normally the *LB*__*Keogh*_ lower bound discussed in Section 4.1.2 builds the envelope around the *query*, a situation we denote *LB_*_*Keogh*_*EQ* for concreteness, and illustrate in Figure 8.*left*. This only needs to be done once, and thus saves the time and space overhead that we would need if we built the envelope around each *candidate* instead, a situation we denote *LB_*_*Keogh*_*EC*.

However, as we show in the next section, we can selectively calculate *LB_*_*Keogh*_*EC* in a “just-in-time” fashion, *only* if all other lower bounds fail to prune. This removes *space* overhead, and as we will see, the *time* overhead pays for itself by pruning more full DTW calculations. Note that in general, *LB_*_*Keogh*_*EQ* ≠ *LB_*_*Keogh*_*EC* and that on average each one is larger about half the time.

#### 4.2.4 Cascading Lower Bounds

**Figure 9:**
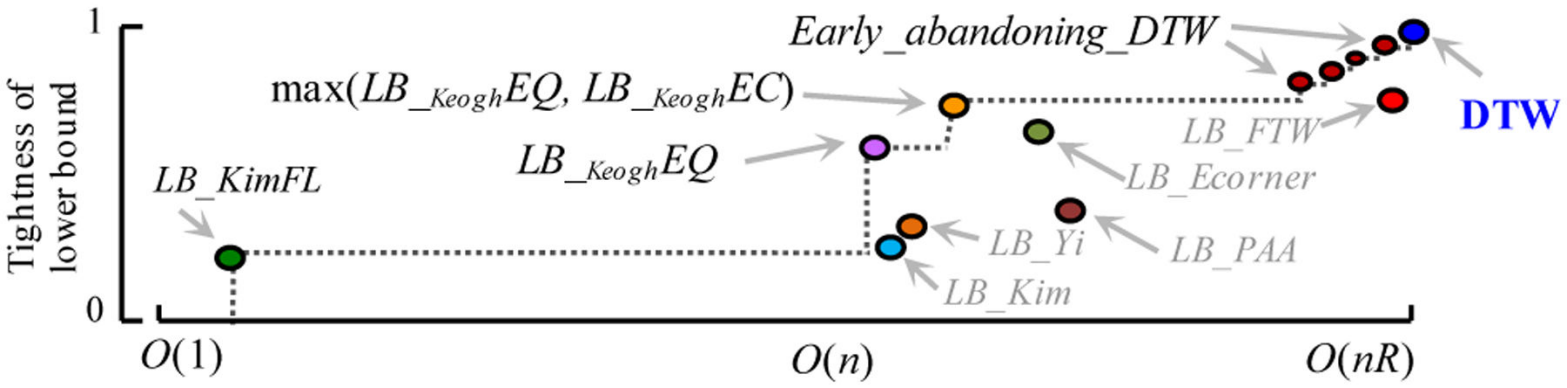
The mean tightness of selected lower bounds from the literature plotted against the time taken to compute them

One of the most useful ways to speed up time series similarity search is the use of lower bounds to admissibly prune off unpromising candidates [6][11]. This has led to a flurry of research on lower bounds, with at least eighteen proposed for DTW [1][6][20][21][33][40][41][42]. In general, it is difficult to state definitively which is the best bound to use, since there is a tradeoff between the tightness of the lower bound and how fast it is to compute. Moreover, different datasets and even different queries can produce slightly different results. However, as a starting point, we implemented all published lower bounds and tested them on fifty different datasets from the UCR archive, plotting the (slightly idealized for visual clarity) results in Figure 9. Following the literature [20], we measured the *tightness* of each lower bound as *LB*(A,B)/*DTW*(A,B) over 100,000 randomly sampled subsequences A and B of length 256.

The reader will appreciate that a *necessary* condition for a lower bound to be useful is for it to appear on the “skyline” shown with a dashed line; otherwise there exists a faster-to-compute bound that is at least as tight, and we should use that instead. Note that the early abandoning DTW discussed in Section 4.1.4 is a special case in that it produces a spectrum of bounds, as at every stage of computation it is incrementally computing the DTW until the last computation gives the final true DTW distance.

Which of the lower bounds on the skyline should we use? Our insight is that we should use *all* of them in a cascade. We first use the *O*(1) *LB_*_*Kim*_*FL*, which while a very weak lower bound prunes many objects. If a candidate is not pruned at this stage we compute the *LB_*_*Keogh*_*EQ*. Note that as discussed in Sections 4.1.3, 4.2.1 and 4.2.2, we can incrementally compute this; thus, we may be able to abandon anywhere between *O*(1) and *O*(*n*) time. If we complete this lower bound without exceeding the *best-so-far*, we reverse the query/data role and compute *LB_*_*Keogh*_*EC* (cf. Section 4.2.3). If this bound does not allow us to prune, we then start the early abandoning calculation of DTW (cf. Section 4.1.4).

Space limits preclude detailed analysis of which lower bounds prune how many candidates. Moreover, the ratios depend on the query, data and size of the dataset. However, we note the following: Detailed analysis *is* available at [43], lesion studies tell us that *all* bounds do contribute to speedup; removing any lower bound makes search at least twice as slow; and finally, using this technique we can prune more than 99.9999% of DTW calculations for a large-scale search.

## 5. EXPERIMENTAL RESULTS

We begin by noting that we have taken extraordinary measures to ensure our experiments are reproducible. In particular, all data and code will be available in perpetuity, archived at [43]. Moreover, the site contains several videos which visualize some of the experiments in real time. We consider the following methods:

- **Naive**: Each subsequence is Z-normalized from scratch. The full Euclidean distance or the DTW is used at each step. Approximately 2/3 of the papers in the literature do (some minor variant of) this.
- **State-of-the-art (SOTA)**: Each sequence is Z-normalized from scratch, early abandoning is used, and the *LB_*_*Keogh*_ lower bound is used for DTW. Approximately 1/3 of the papers in the literature do (some minor variant of) this.
- **UCR Suite**: We use all of our applicable speedup techniques.

DTW uses *R* = 5% unless otherwise noted. For experiments where Naive or SOTA takes more than 24 hours to finish, we terminate the experiments and present the interpolated values, shown in gray. Where appropriate we also compare to an oracle algorithm:

- **GOd’s ALgorithm** (GOAL) is an algorithm that only *maintains* the mean and standard deviation using the online *O*(1) incremental calculations.

It is easy to see that, short of an algorithm that precomputes and stores a *massive* amount of data (quadratic in *m*), GOAL is a lower bound on the fastest possible algorithm for either ED or DTW subsequence search with unconstrained and unknown length queries. The acronym reminds us that we would like to be as close to this *goal* value as possible.

It is critical to note that our implementations of Naive, SOTA and GOAL are incredibly efficient and tightly optimized, and they are not “crippled” in any way. For example, had we wanted to claim spurious speedup, we could implement SOTA recursively rather than iteratively, and that would make SOTA at least an order of magnitude slower. In particular, the code for Naive, SOTA and GOAL is exactly the same code as the UCR suite, except the relevant speedup techniques have been commented out.

While very detailed spreadsheets of all of our results are archived in perpetuity at [43], we present subsets of our results below. We consider wall clock time on a 2 Intel Xeon Quad-Core E5620 2.40GHz with 12GB 1333MHz DDR3 ECC Unbuffered RAM (using just one core unless otherwise explicitly stated).

### 5.1 Baseline Tests on Random Walk

We begin with experiments on random walk data. Random walks model financial data very well and are often used to test similarity search schemes. More importantly for us, they allow us to do reproducible experiments on massive datasets without the need to ship large hard drives to interested parties. We have simply archived the random number generator and the seeds used. We *have* made sure to use a very high-quality random number generator that has a period longer than the longest dataset we consider. In Table 2 we show the length of time it takes to search increasingly large datasets with queries of length 128. The numbers are averaged over 1000, 100 and 10 queries, respectively.

**Figure 10:**
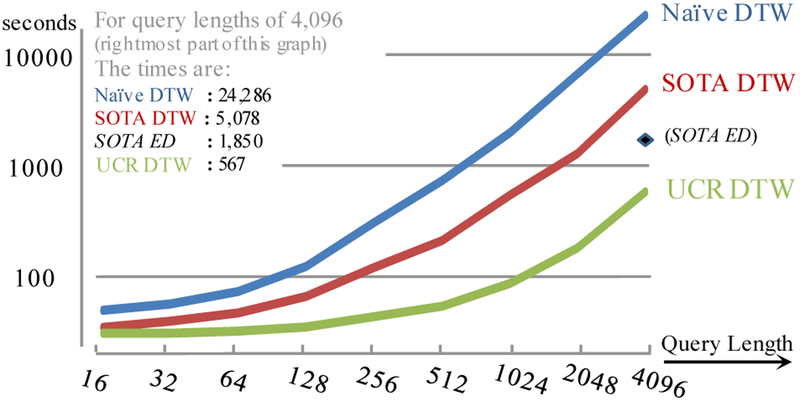
The time taken to search random walks of length 20 million with increasingly long queries, for three variants of DTW. In addition, we include just length 4,096 with SOTA-ED for reference

These results show a significant difference between SOTA and UCR suite. However, this is for a very short query; what happens if we consider longer queries? As we show in Figure 10, the ratio of SOTA-DTW over UCR-DTW *improves* for longer queries.

To reduce visual clutter we have only placed one Euclidean distance value on the figure, for queries of length 4,096. Remarkably, UCR-DTW is even faster than SOTA *Euclidean* distance. As we shall see in our EEG and DNA examples below, even though 4,096 is longer than any published query lengths in the literature, there is a need for even *longer* queries.

It is also interesting to consider the results of the 128-length DTW queries as a ratio over GOAL. Recall that the cost for GOAL is independent of query length, and this experiment is just 23.57 seconds. The ratios for Naive, SOTA and UCR suite are 5.27, 2.74 and 1.41, respectively. This suggests that we are asymptomatically closing in on the fastest possible subsequence search algorithm for DTW. Another interesting ratio to consider is the time for UCR-DTW over UCR-ED, which is just 1.18. Thus, the time for DTW is not significantly different than that for ED, an idea which contradicts an assumption made by almost all papers on time series in the last decade (including papers by the current authors).

### 5.2 Supporting Long Queries: EEG

**Figure 11:**
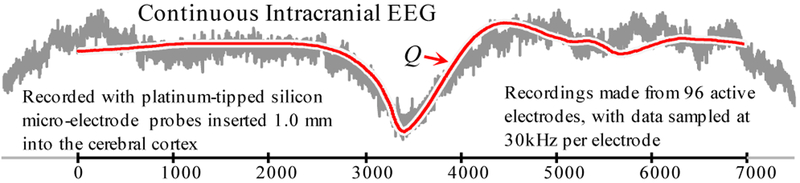
Query *Q* shown with a match from the 0.3 trillion EEG dataset

The previous section shows that we gain the greatest speedup for long queries, and here we show that such long queries are really needed. The first user of the UCR suite was Dr. Sydney Cash, who together with author B.W. wants to search massive archives of EEG data for examples of epileptic spikes, as shown Figure 11.

From a single patient S.C. gathered 0.3 trillion datapoints and asked us to search for a prototypical epileptic spike *Q* he created by averaging spikes from other patients. The query length was 7,000 points (0.23 seconds). Table 3 shows the results.

This data took multiple sessions over seven days to collect, at a cost of approximately $34,000 [43], so the few hours of CPU time we required to search the data are dwarfed in comparison.

### 5.3 Supporting Very Long Queries: DNA

Most work on time series similarity search (and *all* work on time series *indexing*) has focused on relatively short queries, less than or equal to 1,024 data points in length. Here we show that we can efficiently support queries that are two orders of magnitude longer.

We consider experiments with DNA that has been converted to time series. However, it is important to note that we are not claiming any particular bioinformatics utility for our work; it is simply the case that DNA data is massive, and the ground truth can be obtained through other means. As in [35], we use the algorithm in Table 4 to convert DNA to time series^2^.

**Figure 12:**
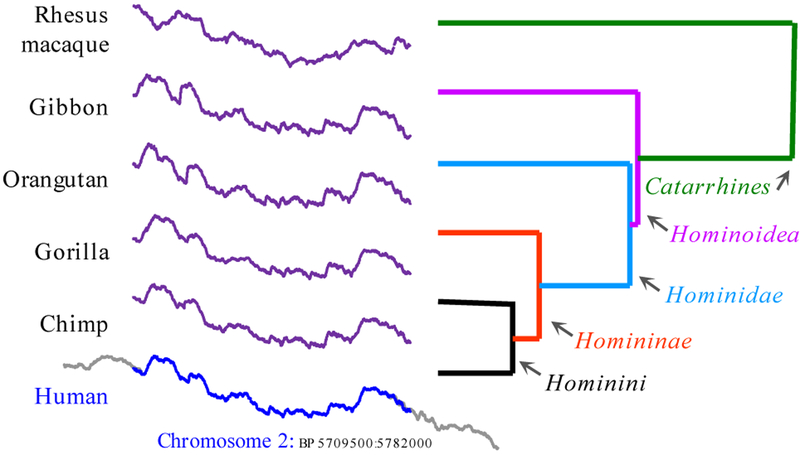
A subsequence of DNA from Human chromosome 2, of length 72,500 beginning at 5,709,500 is clustered using single linkage with its Euclidean distance nearest neighbors from five other primates

We chose a section of Human chromosome 2 (H2) to experiment with. We took a subsequence beginning at 5,709,500 and found its nearest neighbor in the genomes of five other primates, clustering the six sequences with single linkage to produce the dendrogram shown in Figure 12.

Pleasingly, the clustering *is* the correct grouping for these primates [24]. Moreover, because Human chromosome 2 is widely accepted to be a result of an end-to-end fusion of two progenitor ancestral chromosomes 2 and 3 [24], we should expect that the nearest neighbors for the non-human apes come from one of these two chromosomes, and that is exactly what we found.

Our query is of length 72,500, and the genome chimp is 2,900,629,179 base pairs in length. The single-core nearest neighbor search in the entire chimp genome took 38.7 days using Naive, 34.6 days using SOTA, but only 14.6 hours using the UCR suite. As impressive as this is, as we shall show in the next section, we can do *even better*.

#### 5.3.1 Can we do better than UCR Suite?

We claim that for the problem of exact similarity search with arbitrary length queries, our UCR suite is close to optimal. However, it is instructive to consider an apparent counterexample and its simple “patch.”

Consider the search for a query of length 64 considered in Section 5.1. Using GOAL took 9.18 seconds, but UCR suite took only a little longer, just 10.64 seconds. Assume that the original query was:

Q=[2.34, 2.01, 1.99,…]

But we make it three times longer by padding it like this:

QP=[2.34, 2.34, 2.34, 2.01, 2.01, 2.01, 1.99, 1.99, 1.99,…]

Further assume that we do the same to database *T*, to get *TP*, which is three times longer. What can we now say about the time taken for the algorithms? GOAL will take exactly three times longer, and Naive takes exactly nine times longer, because each ED calculation takes three times longer and there are three times as many calculations to do. Our UCR suite does not take nine times longer, as it can partly exploit the “smoothness” of the data; however, its overhead *is* greater than three. Clearly, if we had known that the data was contrived in this manner, we could have simply made a one-in-three downsampled version of the data and query, done the search on this data, and reported the location and distance back in the *TP* space by multiplying each by three.

Of course, this type of pathological contrived data does not occur in nature. However, some datasets are richly *oversampled*, and this has a *very* similar effect. For example, a decade ago, most ECGs were sampled at 256Hz, and that seems to be adequate for virtually all data analysis applications [4]. However, current machines typically sample at 2,048 Hz which, given the above reasoning, would take up to sixty-four times longer to search ((2,048/256)^2^) with almost certainly identical results.

We believe that oversampled data can be searched more quickly by exploiting a provisional search in a downsampled version of the data that can quickly provide a low *best-so-far*, which, when projected back into the original space can be used to “prime” the search by setting a low *best-so-far* at the beginning of the search, thus allowing the early abandoning techniques to be more efficient.

To test this idea, we repeated the experiment in the previous section, with a one-in-ten downsampled version of the chimp genome / human query. The search took just 475 seconds. We denoted the best matching subsequence distance *rD*. We reran the full resolution search after initializing the *best-so-far* to rD*10. This time the search fell from 14.64 hours to 4.17 hours, and we found the same answer, as we logically must.

Similar ideas have been proposed under the name of Iterative Deepening DTW [1] or Multi Scale DTW [27][42]; thus, we will not further develop this idea here. We simply caution the reader that oversampled (i.e., “smooth”) data may allow more speedup than a direct application of the UCR suite may initially suggest.

### 5.4 Realtime Medical and Gesture Data

The proliferation of inexpensive low-powered sensors has produced an explosion of interest in monitoring real time streams of medical telemetry and/or Body Area Network (BAN) data [22]. There are dozens of research efforts in this domain that explicitly state that while monitoring under DTW is *desirable*, it is impossible [38]. Thus, approximations of, or alternatives to DTW are used. Dozens of suggested workarounds have been suggested. For example, [16] resorts to only “*dealing with shorter test and class templates, as this is more efficient*”; many research efforts including [36] resort to a low cardinality version of DTW using integers, or DTW approximations that operate on piecewise linear approximations of the signals [20][29], or drastically downsampled versions of the data [12][30]. In spite of some progress from existing ideas such as lower bounding, [3] bemoans DTW is “*still too slow for gesture recognition systems*”, [29] laments that the “*problem of searching with DTW (is) intractable*”, [12] says “*Clearly (DTW) is unusable for real-time recognition purposes*” and [34] notes “*Processing of one hour of speech using DTW takes a few hours*.”

We believe that the UCR suite makes all of these objections moot. DTW *can* be used to spot gestures/brainwaves/musical patterns/anomalous heartbeats in *real-time*, even on low-powered devices, even with multiple channels of data, and even with multiple simultaneous queries.

To see this, we created a dataset of one year of electrocardiograms (ECGs) sampled at 256Hz. We created this data by concatenating the ECGs of more than two hundred people, and thus we have a highly diverse dataset, with 8,518,554,188 datapoints. We created a query by asking USC cardiologist Dr. Helga Van Herle to produce a query she searches for on a regular basis, she created an idealized Premature Ventricular Contraction (PVC). The results are shown in Table 5. While this was on our multi-core desktop machine, the fact that our results are 29,219 times faster than real-time (256Hz) suggests that real-time DTW is tenable even on low-power devices.

### 5.5 Speeding up Existing Mining Algorithms

In this section, we demonstrate that we can speed up much of the code in the time series data mining literature with minimal effort, simply by replacing their distance calculation subroutines with the UCR suite. In many cases, the difference is small, because the algorithms in question already typically try to prune as many distance calculations as possible. As an aside, in at least some cases we believe that the authors could benefit from redesigning the code in light of the drastically reduced cost for similarity search that UCR suite offers. Nevertheless, even though the speedups are relatively small (1.5X to 16X), they are “free”, requiring just minutes of cut-and-paste code editing.

**Time Series Shapelets** have garnered significant interest since their introduction in 2009 [39]. We obtained the original code and tested it on the Face (four) dataset, finding it took 18.9 minutes to finish. After replacing the similarity search routine with UCR suite, it took 12.5 minutes to finish.

**Online Time Series Motifs** generalize the idea of mining repeated patterns in a batch time series to the streaming case [25]. We obtained the original code and tested it on the EEG dataset used in the original paper. The fastest running time for the code assuming linear space is 436 seconds. After replacing the distance function with UCR suite, it took just 156 seconds.

**Classification of Historical Musical Scores** [10]. This dataset has 4,027 images of musical notes converted to time series. We used the UCR suite to compute the rotation-invariant DTW leave-one-out classification. It took 720.6 minutes. SOTA takes 142.4 hours. Thus, we have a speedup factor of 11.8.

**Classification of Ancient Coins** [15]. 2,400 irregularly shaped coins are converted to time series of length 256, and rotation-invariant DTW is used to search the database, taking 12.8 seconds per query. Using the UCR suite, this takes 0.8 seconds per query.

**Clustering of Star Light Curves** is an important problem in astronomy [20], as it can be a preprocessing step in outlier detection [31]. We consider a dataset with 1,000 (purportedly) phase-aligned light curves of length 1,024, whose class has been determined by an expert [31]. Doing spectral clustering on this data with DTW (*R* = 5%) takes about 23 minutes for all algorithms, and averaged over 100 runs we find the Rand-Index is 0.62. While this time may seem slow, recall that we must do 499,500 DTW calculations with relatively long sequences. As we do not trust the original claim of phase alignment, we further do *rotation-invariant* DTW that dramatically increases Rand-Index to 0.76. Using SOTA, this takes 16.57 days, but if we use the UCR suite, this time falls by an order of magnitude, to just 1.47 days on a single core.

## 6. DISCUSSION AND CONCLUSIONS

While our work has focused on fast *sequential search*, we believe that for DTW, our work is faster than all known *indexing* efforts. Consider [2], which indexes a random walk time series of length 250,000 to support queries of length 256. They built various indexes to support DTW queries, noting that the fastest of the four carefully tuned approaches requires approximately 15,000 pages accesses to answer a query. These disk accesses are necessarily *random* accesses. While they did not give wall clock time, if we assume an HDD spindle speed of 7,200 rpm (average rotational latency = 4.17*ms*), then just the disk I/O time to answer this query must be at least 62.55 seconds. However, as we have shown, we can load all of the data into the main memory with more efficient sequential disk accesses and answer these queries in 0.4 seconds, *including* disk I/O time, on a single core machine.

Note that *all* experiments in this paper include the time taken to read the data from disk. However, for more than a few million objects this time is inconsequential thus we did not report it separately.

We have made a strong and unintuitive claim in the abstract. We said that our UCR-*DTW* is faster than all current *Euclidean* distance searches. In Table 5, for example, we show that DTW can be three times faster than state-of-the-art ED searching. How is this possible? Recall that all Euclidean searches in the literature require an *O*(*n*) data normalizing step to be performed for each subsequence. Thus, no matter how effective the pruning/search strategy used, the amortized time for a single sequence must be at least *O*(*n*). In contrast, using the ideas developed in this work, the vast majority of potential DTW calculations are pruned with *O*(1) work, while some require up to *O*(*n*) work, and only a vanishingly small fraction require *O*(*nR*) work. The weighted average of these possibilities is less than *O*(*n*).

To put our results in perspective, we compare them with a very recent state-of-the art embedding-based DTW search technique, called EBSM (*including* the variant called BSE) [28]. This is an excellent paper to use as a benchmark, as it exhaustively compares to almost all other methods in the literature, and it tests its contributions over different datasets, query lengths, warping widths, etc. In contrast to EBSM:

- Our method is *exact*; EBSM is *approximate*.
- EBSM requires setting some parameters (number of reference sequences, dimensionality, number of split points, etc.). Our method requires *zero* parameters.
- EBSM requires offline preprocessing that takes over 3 hours for just 1 million objects. We have *zero* preprocessing time.
- The EBSM method does not, and cannot, Z-normalize. As noted in Section 1.2.1, we believe that Z-normalizing is critical, and we have shown that failure to do it hurts on 45 out of 45 of the UCR time series classification datasets.
- EBSM can support queries in a predetermined range, which must be predetermined and limited for efficiently. In contrast, we have no minimum/maximum query length.
- We can also handle exact queries under uniform scaling [11].
- Finally, we are simply much faster! (c.f. Section 1)

Note, however, that there *can* be great utility in fast approximate search. There exist data mining algorithms that can use a combination of (hopefully few) exact distance measures and (hopefully much faster) approximate searches to produce overall exact results [35]. However an approximate search method being faster than our approach is a very high threshold to meet.

We have shown our suite of ideas is 2 to 164 times faster than the *true* state-of-the-art, depending on the query/data. However, based on the quotes from papers that we have sprinkled throughout this work, we are sometimes more than 100,000 times faster than recent papers; how is this possible? The answer seems to be that it is possible to produce very naive implementations of DTW. For example, the *recursive* version of DTW can be one to three orders of magnitude slower than the *iterative* version, depending on the computer language and query length. Thus, the contributions of this paper are twofold. First, we have shown that much of the recent pessimism about using DTW for real-time problems was simply unwarranted [6]. *Existing* techniques, especially lower bounding, if carefully implemented can make DTW tractable for many problems. Our second contribution is the introduction of the UCR suite of techniques that make DTW and Euclidean distance subsequence search significantly faster than current state-of-the-art techniques.

We regret that the page limitations preclude full pseudo-code; however, full pseudo-code (and source-code) *is* available at [43].

In future work we plan to revisit algorithms for time series motif discovery [25][26], anomaly detection [35][31], time series summarization, shapelet extraction [39], clustering, and classification [6] in light of the results presented in this work.

## Figures and Tables

**Table 1:** Subsequence search with online Z-normalization

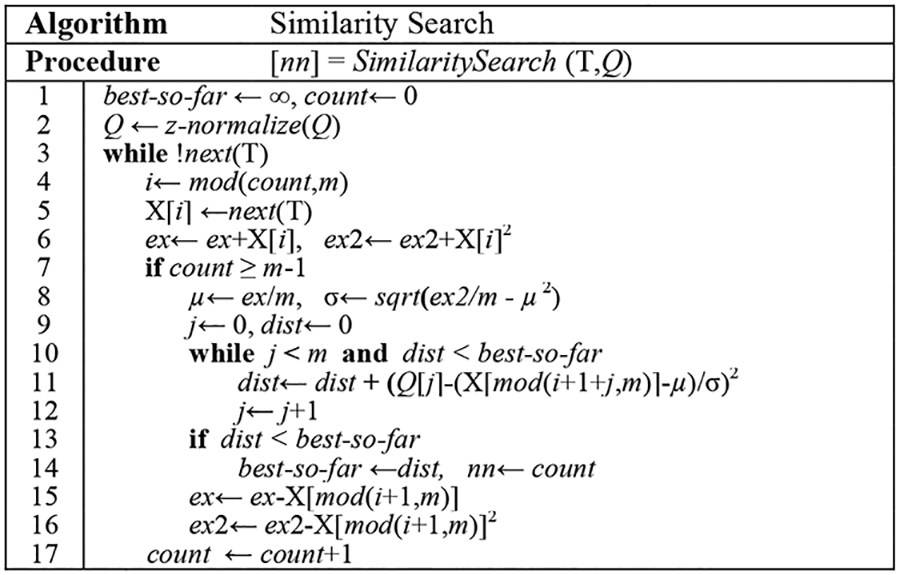

**Table 2:** Time taken to search a random walk dataset with |*Q*| =128

	Million (*Seconds*)	Billion (*Minutes*)	Trillion (*Hours*)
**UCR-ED**	0.034	0.22	3.16
**SOTA-ED**	0.243	2.40	39.80
**UCR-DTW**	0.159	1.83	34.09
**SOTA-DTW**	2.447	38.14	472.80

**Table 3:** Time to search 303,523,721,928 EEG datapoints, |*Q*| = 7000

Note that only ED is considered here because DTW may produce false positives caused by eye blinks		**UCR-ED**	**SOTA-ED**
	**EEG**	3.4 hours	494.3 hours

**Table 4:** An algorithm to convert DNA to time series

T_1_ = 0,	**for** i = 1	**to** |DNAstring|	
		**if** DNAstring_i_ = A,	**then** T_i+1_ = T_i_ + 2
		**if** DNAstring_i_ = G,	**then** T_i+1_ = T_i_ + 1
		**if** DNAstring_i_ = C,	**then** T_i+1_ = T_i_ − 1
		**if** DNAstring_i_ = T,	**then** T_i+1_ = T_i_ − 2

**Table 5:** Time taken to search one year of ECG data with |*Q*| = 421

	UCR-ED	SOTA-ED	UCR-DTW	SOTA-DTW
**ECG**	4.1 minutes	66.6 minutes	18.0 minutes	49.2 hours

## Footnotes

1 This collection of sizes is very optimistic. The step size should be at most 100, creating two orders of magnitude space overhead.

2 To preserve the reversible one-to-one mapping between time series and DNA we normalize the offset by subtracting *round*(*mean*) and we do not divide by the STD.
